# Supplementary material for: Photonic crystal cavities from hexagonal boron nitride
Source: Nat Commun. 2018 Jul 5;9:2623. doi: 10.1038/s41467-018-05117-4 (PMC6033931; doi:10.1038/s41467-018-05117-4)
Supplement: Supplementary file 1 — Supplementary Information [file 41467_2018_5117_MOESM1_ESM.pdf]

# Supplementary Information

# Photonic Crystal Cavities from hexagonal Boron Nitride

Sejeong Kim[1,#,*], Johannes E. Fröch[1,#], Joe Christian[2], Marcus Straw[2], James Bishop[1], Daniel Totonjian[1], Kenji Watanabe[3], Takashi Taniguchi[3], Milos Toth[1,*], Igor Aharonovich[1,*]

*[1]Institute of Biomedical Materials and Devices (IBMD), Faculty of Science, University of Technology Sydney, Ultimo, NSW, 2007, Australia*

*[2]Thermo Fisher Scientific, 5350 NE Dawson Creek Drive, Hillsboro, OR 97214-5793 USA*

*[3]National Institute for Materials Science, 1-1 Namiki Tsukuba Ibaraki 305-0044 Japan*

*correspondence to Sejeong.Kim-1@uts.edu.au, milos.toth@uts.edu.au, igor.aharonovich@uts.edu.au

# These authors contributed equally to this work.

## Supplementary Note 1. Thickness and smoothness of suspended hBN flakes

Exfoliated hBN films on trenched substrates are strong enough to withstand sagging or wrinkling, as shown by the SEM images in Supplementary Figure 1 obtained directly after exfoliation. Film thicknesses in the range of 200 nm – 600 nm and smooth morphologies allow for the fabrication of optical cavities.

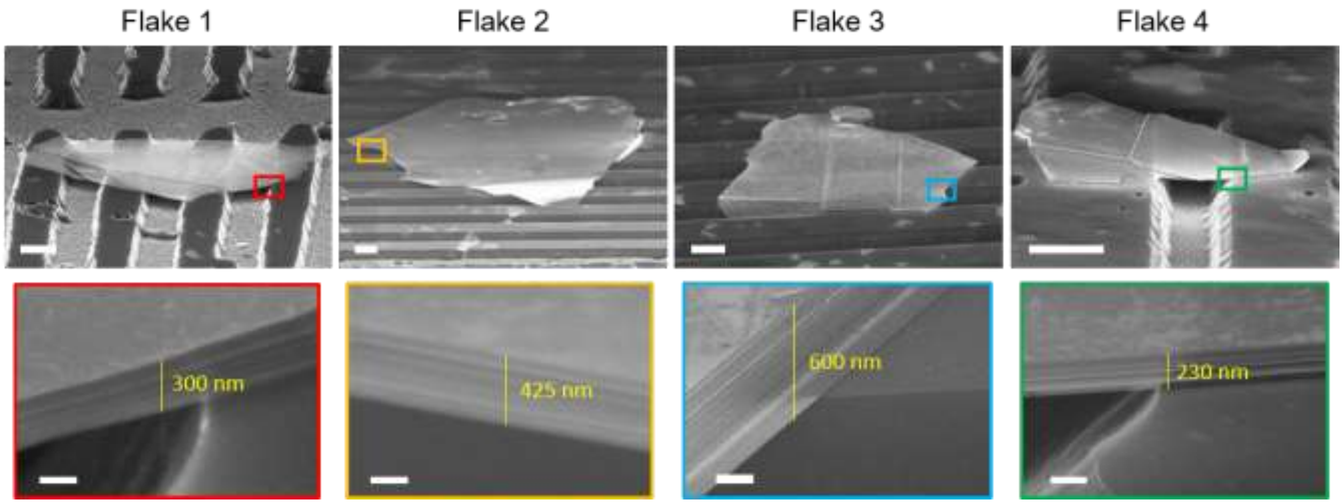


**Supplementary Figure 1 Films of hexagonal Boron Nitride after exfoliation from a bulk crystal.** The flakes of hBN are mechanically stable, appear widely smooth with no wrinkling. Flakes show thicknesses in the range of 200 nm - 600 nm. Scale bars in the upper row images correspond to 10 µm. Scale bars in the lower row images correspond to 200 nm.

## Supplementary Note 2. Fabrication of cavities in hBN by RIE – EBIE

The fabrication of hBN cavities via the RIE – EBIE approach is illustrated schematically in Supplementary Fig. 2. A flake of hBN on a trenched substrate before all fabrication steps is shown in Supplementary Fig. 2b. An optical microscope image of a patterned area is shown in Supplementary Fig. 2c. The same area in a SEM image in top and tilted view after all processing steps are shown in Supplementary Fig. 2d and 2e.

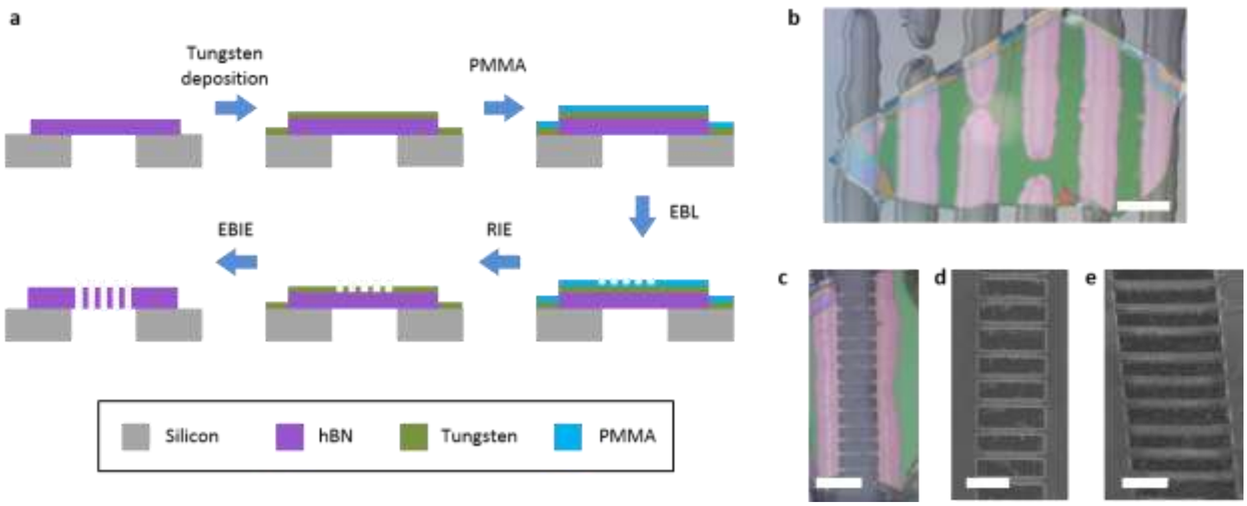


**Supplementary Figure 2 Fabrication of hexagonal Boron Nitride photonic crystal cavities. a,** Schematic illustration of the workflow used to fabricate suspended cavities in hBN. hBN (purple) is exfoliated onto a trenched Si Substrate (grey). Then, a 15 nm tungsten layer is deposited. PMMA is spun on. In the next step, the cavity designs are patterned into PMMA via EBL, and subsequently transferred into the tungsten mask via RIE. Side walls and residual hBN are etched by EBIE, finally the tungsten mask is removed using $H_2O_2$. **b,** A hBN flake before all fabrication steps, scale bar corresponds to 20 µm. **c,** Part of the same flake after all fabrication steps, scale bar corresponds to 10 µm. **d,** Top view and **e,** tilted view of several cavities in hBN, scale bar corresponds to 5 µm in both cases.

## Supplementary Note 3. Comparison of etching methods

In this paper, we first demonstrated nanofabrication of hBN photonic crystal cavities. Here, we show a comparison of the investigated hBN etching methods, summarized in Supplementary Table 1, and address the advantages and drawbacks of each technique.

**Supplementary Table 1 Comparison between reactive ion etching (RIE), focused ion beam (FIB) etching, and electron beam induced etching (EBIE).**

| Etching method | RIE | FIB | EBIE |
|---|---|---|---|
| Mask-free | No | Yes | Feasible [Supplementary Note 7] |
| Crystal damage [Supplementary Note 6] | No | Yes | No |
| Etching speed | Fast, large area | Slow, small area | Slow, small area |
| Post annealing required | No | Yes | No |
| Sidewall slope [Supplementary Note 5] | Can be vertical after optimization | Inherent slope | Vertical without optimization |
| Sidewall roughness | Rough | Smooth | Rough |
| Selective tuning | No | Yes | Yes |
| Effect on emitter | No | Yes | Unknown |

## Supplementary Note 4. Cavity design and theoretical Q-factor

FDTD simulations were performed using the Lumerical software package. Our 1D photonic crystal cavities each have 25 uniform-sized air holes, as is shown in Supplementary Fig. 3a. The cavity is formed by modulating lattice constants in the middle of the structure. The region with modulated lattice constant is called a photonic well, while the region with a periodic lattice constant is called a photonic mirror. The lattice constant in the photonic well decreases gradually in order to avoid an abrupt change, as is shown in Supplementary Fig. 3b. A 1D photonic crystal with lattice constant (*a*) having $h_x$=130 nm, $h_y$=550 nm, *t*=230 nm, *w*=750 nm has 4 prominent modes in the visible wavelength range. F0, F1, and F2 are modes from the first dielectric band, while S0 is the mode from the second dielectric band. The frequencies of the fundamental mode and higher order modes are determined by the photonic band used for the cavity design. In our case, the lowest energy photonic band forms a photonic well, the fundamental mode has the highest energy, and is therefore located at the shortest wavelength[1]. We note that the S0 mode is not the air mode because cavities designed to form a photonic well from the air mode are different from those employed here, i.e. lattice constants in the photonic well region should be larger than in the photonic mirror region. We additionally conducted simulations to see how nanobeam thickness and width affect the *Q*-factor. First, we fixed all the parameter to *a*=250 nm, $h_x$=130 nm, $h_y$=550 nm, *w*=750 nm, and spanned the thickness from 150 nm to 350 nm. Within this range, larger thickness provides higher Q-factor. Next, we fix thickness to 300 nm and vary nanobeam width (*w*). Q-factor is maximum when the width of the 1D PCC is 760 nm.

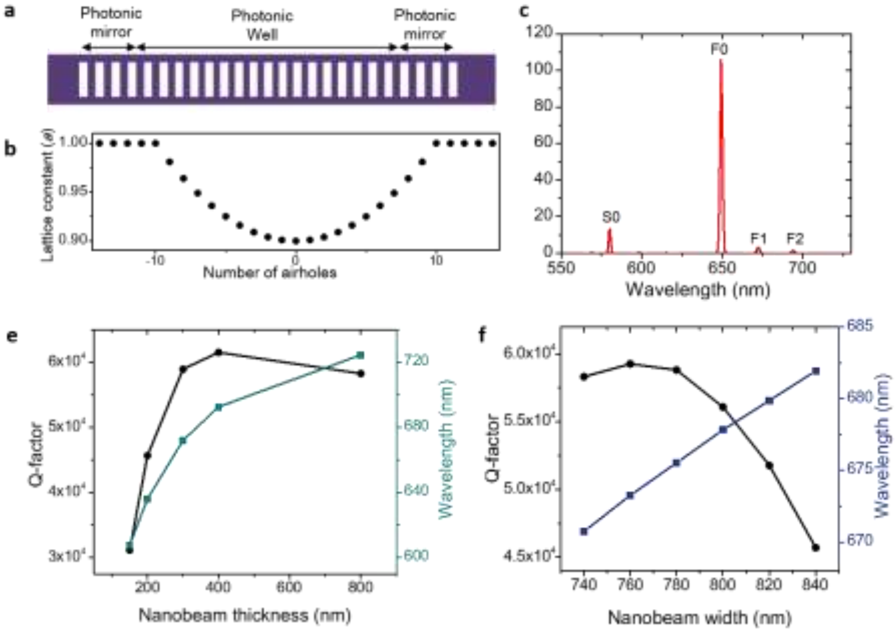


**Supplementary Figure 3 3D FDTD modelling for 1D photonic crystal cavity**. **a,** Schematic showing 1D ladder-like photonic crystal cavity. **b,** Photonic mirror region has lattice constant of '*a*', while photonic well region has modulated lattice constant as plotted in the graph. **c,** Spectrum showing the S0, F0, F1, and F2 modes. **d,** Q-factor with respect to thickness of nanobeam. **e,** Q-factor with respect to nanobeam width (*w*).

## Supplementary Note 5. Sidewall slopes

We compare sidewall slope and roughness caused by the various etching methods. Supplementary Fig. 4a and Fig. 4b show slanted sidewalls after RIE etching. In the case of other material systems, it has been shown that RIE etching can yield vertical sidewalls once conditions are optimized, including the gas flow rate and gas mixture rate. This has, however, not been achieved to date with hBN. In hBN, cavities fabricated by FIB milling show more vertical sidewalls compared to RIE. But, due to the beam shape and material re-deposition that are typical of FIB, slanted sidewalls and blunt edges were obtained. On the other hand, if we use masked EBIE as described above, almost-vertical sidewalls are achieved with a slant of less than 3° for the first 200 nm etching depth and less than 6° beneath, indicated by the two white dashed lines shown in Supplementary Fig. 5d. However, the sidewall roughness is greater than in the case of FIB and thus limits the *Q*-factors of cavities fabricated to date by EBIE. However, as EBIE of hBN is a recent technique, we expect that further refinement of the process will yield improved performance. EBIE therefore has the potential to achieve higher *Q*-factors, as it is not limited by the crystal damage observed in samples fabricated by FIB milling.

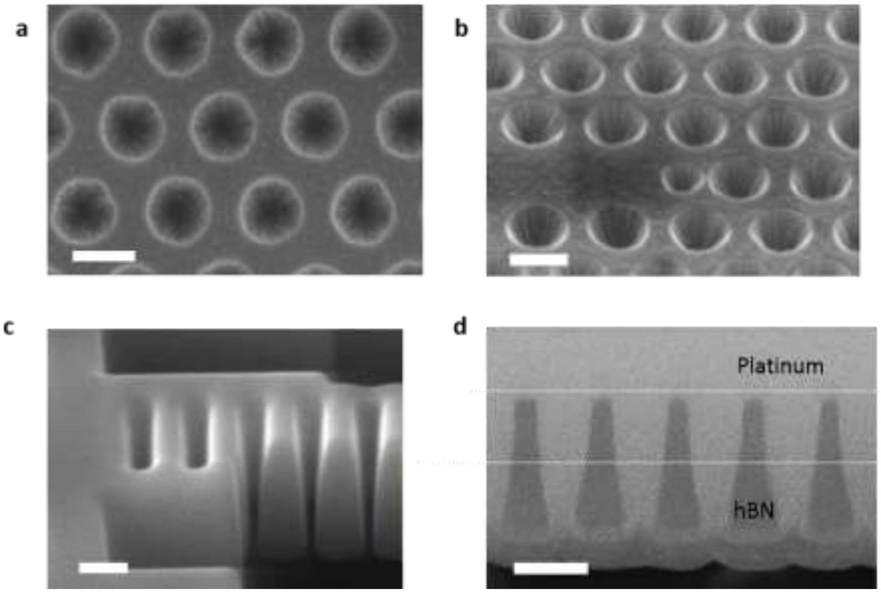


**Supplementary Figure 4 Sidewall slopes. a,** Top-view SEM image after RIE. **b,** Tilted (45º) view after RIE. **c,** Tilted view (52º) of a cross section after FIB etching. **d,** Tilted view (60º) of a cross section after EBIE. Scale bars in all images correspond to 200 nm.

## Supplementary Note 6. Crystalline damage after fabrication

Crystalline damage after all processing steps (including annealing of FIB cavities) was analyzed by Raman spectroscopy. Spectra of pristine hBN and areas containing photonic crystals pattern by EBIE and FIB are compared. Raman analysis shows that there is no obvious crystalline structure damage after the conducted fabrication steps using EBIE, evidenced by no change in the center position and FWHM of the characteristic $E_{2g}$ phonon mode at 1366 cm$^{-1}$ (Supplementary Fig. 5a). Yet, for a cavity fabricated by FIB, even after annealing, crystal damage remains, as can be seen by an increase in the FWHM.

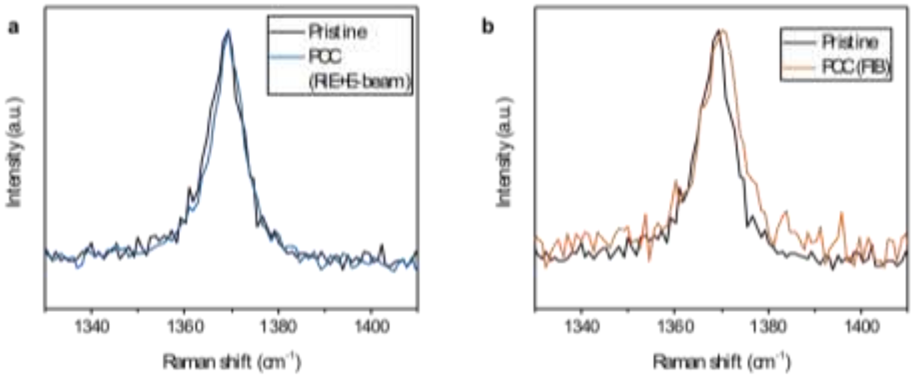


**Supplementary Figure 5 Crystal damage.** Comparison of Raman spectra of pristine hBN (black) with **a,** RIE/ EBIE-processed area (blue) and **b**, FIB-processed area (orange).

## Supplementary Note 7. Mask-free EBIE fabrication

FIB is a well-known, conventional direct-write milling technique. We also investigated the feasibility of direct-write fabrication of cavities using the less common EBIE technique, illustrated schematically in Supplementary Fig. 6a. This process is driven by site specific chemical reactions between a precursor ($H_2O$) and hBN, induced by the electron beam, as is illustrated in Supplementary Fig. 6b. The process is minimally-invasive in that it limits the damage to the surrounding material. An example of a structure fabricated entirely by direct-write EBIE is shown in Supplementary Fig. 6c. We note that the process shown in Supplementary Fig. 2a was used to fabricate the cavities presented in this work because it eliminated two phenomena – a trade-off between electron beam energy and spatial resolution that is inherent to direct-write EBIE, and the effects of electron beam (and specimen stage) drift on structure geometry.

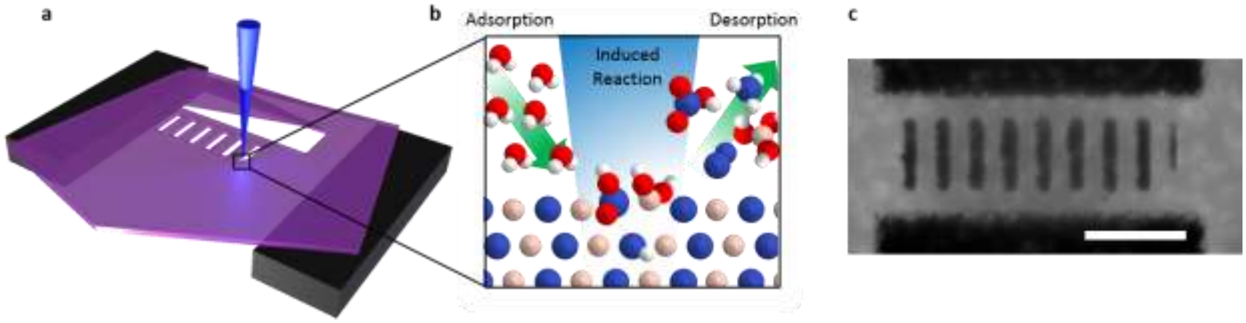


**Supplementary Figure 6 Direct-write fabrication of cavities in hBN by EBIE. a**, Schematic of a suspended flake of hBN on a trenched silicon substrate. A focused electron beam is scanned to etch a structure into the material directly, without an etch mask. **b**, On an atomic scale the beam induces chemical reactions (oxygen: red, hydrogen; white, nitrogen: blue, boron: beige). Water molecules delivered to the reaction site undergo beam induced reactions with hBN. **c**, Example of a cavity test structure fabricated using EBIE. Scale bar corresponds to 1 µm.

## Supplementary Note 8. Comparison of cavity *Q*-factors to other systems hosting color centers

**Supplementary Table 2 *Q*-factors from diverse cavities.** Comparison of cavities and their *Q*-factors with systems hosting color centers similar to hBN.

| Material | hBN | Diamond | 3C SiC | 4H SiC | YSO | GaN |
|---|---|---|---|---|---|---|
| Refractive Index | 1.8 | 2.4 | 2.6 | 2.6 | 1.8 | 2.5 |
| Fabrication method | RIE/EBIE, FIB | FIB, RIE | RIE | RIE | FIB | RIE |
| *Q* (exp) | 1700, 2100 | 700 ($\lambda$=810 nm)[2] 11000 ($\lambda$=734nm)[3] | 1500 ($\lambda$=1128)[4] | 6700 ($\lambda$=700)[5] | 3000 ($\lambda$=596)[6] | 5200 ($\lambda$=454)[7] |
| Mode Volume | 1.39 | 0.7, 0.52 | 0.9 | 0.5 | 1 | 1.7 |
| quantum emitters | Yes | Yes | No | Yes | No | Yes |
| Cavity Type | 1D | 1D | 2D | 1D | 1D | 1D |

## Supplementary Note 9. Emitters created in the patterned area

Here, we used a different flake from the one shown in Figure 4 of the main manuscript to illustrate the fact that emitters are observed after processing of hBN all flakes investigated in this study. We also included a detailed analysis on the emitter designated 'SPE1', showing photostability (532 cw laser, 30 μW), lifetime, and antibunching data.

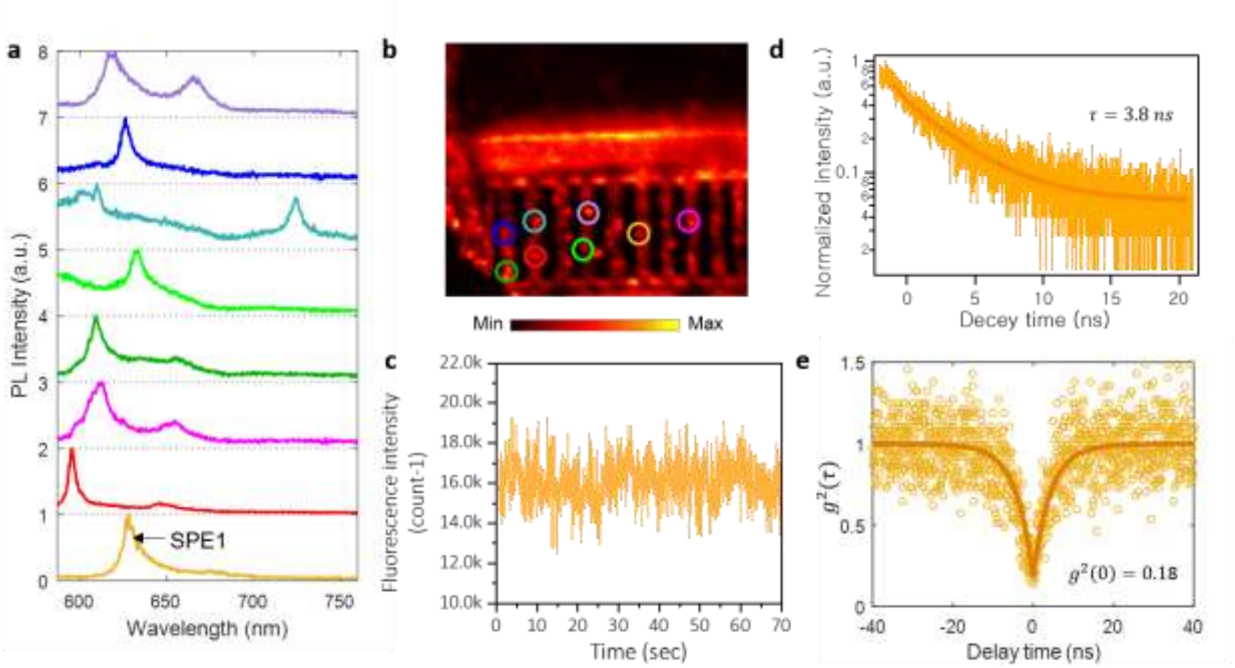


**Supplementary Figure 7 Generation of emitters within patterned area**. **a,** Spectra of emitters from the patterned area. **b, a** PL map of the flake with colored circles showing positions of emitters. **c,** Fluorescence intensity as a function of time demonstrating the photostability of emitter 'SPE1'. **d,** Lifetime measurement of SPE1 showing an excited-state lifetime of 3.8 ns. **e.** Antibunching curve with a $g^2(0)$ value of 0.18.

## Supplementary Note 10. Optical Measurement Setup

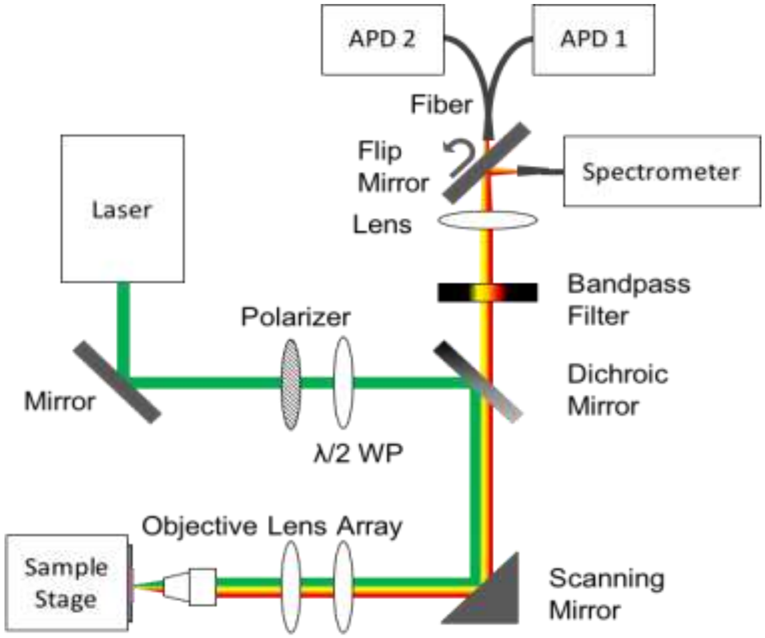


**Supplementary Figure 8 Schematic of the setup for optical measurement**. Light (532 nm) of a laser is guided through a Polarizer and $\lambda/2$ Wave Plate (WP), reflected by a scanning mirror, allowing for scanning, focused through an objective onto the sample. Light is collected by the same objective, passes the dichroic mirror, guided through a bandpass filter and focused via a lens onto a flip mirror and either coupled into a fiber guiding into a spectrometer, or a Hanburry-Brown and Twiss Setup for determination of the quantum nature of emitters.

## References

1. Ahn, B.-H. et al. One-dimensional parabolic-beam photonic crystal laser. *Opt. Express* **18**, 5654-5660 (2010).
2. Riedrich-Moller, J. et al. One- and two-dimensional photonic crystal microcavities in single crystal diamond. *Nature Nanotechnology* **7**, 69-74 (2012).
3. Burek, M.J. et al. High quality-factor optical nanocavities in bulk single-crystal diamond. *Nature Communications* **5**, 5718 (2014).
4. Calusine, G., Politi, A. & Awschalom, D.D. Silicon carbide photonic crystal cavities with integrated color centers. *Applied Physics Letters* **105**, 011123 (2014).
5. Bracher, D.O. & Hu, E.L. Fabrication of High-Q Nanobeam Photonic Crystals in Epitaxially Grown 4H-SiC. *Nano Letters* **15**, 6202-6207 (2015).
6. Zhong, T., Rochman, J., Kindem, J.M., Miyazono, E. & Faraon, A. High quality factor nanophotonic resonators in bulk rare-earth doped crystals. *Optics Express* **24**, 536-544 (2016).
7. Rousseau, I. et al. Quantification of scattering loss of III-nitride photonic crystal cavities in the blue spectral range. *Physical Review B* **95**, 125313 (2017).
